# Factors influencing surgical performance and learning progress in minimally invasive surgery – results of an interdisciplinary multicenter study

**DOI:** 10.1097/JS9.0000000000000590

**Published:** 2023-07-14

**Authors:** Johannes Ackermann, Jorun Baumann, Julian Pape, Julia Pahls, Zino Ruchay, Carolin Spüntrup, Bernd Holthaus, Günter Noé, Michael Anapolski, Ivo Meinhold-Heerlein, Göntje Peters, Damaris Willer, Anna Westermann, Sandra Brügge, Veronika Günther, Nicolai Maass, Liselotte Mettler, Ibrahim Alkatout

**Affiliations:** a Kiel School of Gynaecological Endoscopy, Department of Gynaecology and Obstetrics, University Hospitals Schleswig-Holstein, Campus Kiel, Kiel; b Pelvic School Saarbrücken, Saarbrücken; c Clinic of Obstetrics and Gynecology, St. Elisabeth Hospital, Damme; d Department of Obstetrics and Gynecology, University Witten/Herdecke, Rheinland Klinikum Dormagen, Dormagen; e Department of Gynecology and Obstetrics, University Hospital of Giessen, Giessen, Germany

**Keywords:** laparoscopic surgery, laparoscopic training, learning curve, surgical education, surgical talent

## Abstract

**Background:**

Advancing surgical techniques require a high level of adaptation and learning skills on the part of surgeons. The authors need selection procedures and decision support systems for the recruitment of medical students and young surgeons. The authors aimed to investigate factors influencing the surgical performance and learning abilities of surgeons and medical students.

**Materials and methods:**

The training scores of persons attending 16 standardized training courses (at three training centers) of the German Working Group for Gynecological Endoscopy (AGE e.V.) from 2017 to 2020, individual characteristics, and the results of psychomotor tests of three-dimensional imagination and hand–eye coordination were correlated. Similar analyses were performed for medical students in their final clinical year from 2019 to 2020. The training concept was evaluated in a prospective, multicenter, interdisciplinary, multinational setting.

**Results:**

In all, 180 of 206 physicians (response rate 87.4%) and 261 medical students (response rate 100%) completed the multistage training concept successfully. Of personal characteristics, the strongest correlation was noted for good surgical performance and learning success, and the absolute number of performed laparoscopic surgeries (*r*=0.28–0.45, *P*<0.001/*r*=0.1–0.28, *P*<0.05). A high score on the spatial visualization ability test was also correlated with good surgical performance (*r*=0.18–0.27, *P*<0.01). Among medical students with no surgical experience, however, age was negatively correlated with surgical performance, that is the higher the age, the lower the surgical performance (*r*=0.13/*r*=0.22, *P*<0.05/*P*<0.001).

**Conclusion:**

Individual factors (e.g. surgical experience, self-assessment, spatial visualization ability, eye–hand coordination, age) influence surgical performance and learning. Further research will be needed to create better decision support systems and selection procedures for prospective physicians. The possibilities of surgical training should be improved, promoted, and made accessible to a maximum number of surgical trainees because individual learning curves can be overcome even by less talented surgeons. Training options should be institutionalized for those attending medical school.

## Introduction

Highlights

- In laparoscopic surgery, new surgical techniques and procedures demand a high level of skill in terms of adaptation and learning.
- However, the essential personal characteristics, skills, and experience of surgeons remain unclear.
- We performed correlation analyses of general characteristics, spatial visualization abilities, eye–hand coordination, and previous laparoscopic experience to assess factors influencing surgical performance on the pelvitrainer.
- We found that prior experience as well as spatial visualization ability and eye–hand coordination had a substantial impact on surgical performance.
- Further research will be needed to determine whether specific tests and selection procedures can be used to select or guide prospective surgeons.

Surgical techniques in medicine are subject to ongoing development^1^. Especially in the era of minimally invasive and robotic-assisted surgery, surgeons are constantly exposed to new techniques and surgical procedures, which signify new training options^2^ and require a high level of skill in terms of adaptation and learning. This is contrasted by the absence of institutionalized training, the failure to identify potential surgeons in medical school and establish individualized funding programs^3^. Furthermore, a good surgeon is usually defined by his/her perception of skills rather than factual knowledge^4,5^.

The qualities of a good surgeon include manual dexterity, clinical understanding, and empathy^6^. However, the personal characteristics, skills, and experience actually needed to achieve these qualities or facilitate their achievement remain unclear. Evidence-based studies on this subject are rare. We lack decision support systems and selection programs for prospective surgeons. Consequently, the young physician who will receive the opportunity to train as a surgeon remains a matter of chance. Many physicians discover their suitability for a surgical specialty by trial and error. In the worst case, this never happens. In the interest of medical ethics, health economics as well as individual physicians, we need screening methods that would indicate a doctor’s suitability for the surgical profession at an early stage.

A well-known factor that determines the success of surgery and associated complication rates, independent of all other factors, is the surgeon’s experience^7–9^. However, we have no unequivocal means of measuring or defining experience. Options include the absolute number of operations, absolute operating time, or the average operating time over a certain period. We also need answers to a series of questions: *What factors enable individual surgeons to achieve a faster learning curve and better surgical performance with, for example, fewer procedures than others? Do surgical training courses compensate surgery on real patients? Can less talented surgeons compensate for their deficit by means of adequate training? If yes, what should the training consist of?*

Based on our recent work on surgical education and training^10–14^, the aim of the current report was to investigate whether specific personal characteristics and practical skills of surgeons and medical students could influence surgical performance and learning progress in minimally invasive surgery. To the best of our knowledge, this is the first prospective, multicenter interdisciplinary investigation of factors influencing the surgical performance of surgeons and medical students in a standardized, objective, and reproducible training setting.

## Materials and methods

The experimental training concept was offered in three settings: (1) An international group of physicians attending 16 standardized training courses of the German Gynecological Endoscopy Working Group (AGE e.V.), held in English and German, were offered the opportunity to participate in the study. The courses were conducted at three different training centers (anonymized) in Germany, from 2017 to 2020. (2) The training course was also offered to general surgeons and urologists in an interdisciplinary setting at a University Hospital in Germany (anonymized). (3) Furthermore, we integrated the same training course into the gynecology internship of students in their fifth year of medical school of a University Hospital in Germany (anonymized). Data were collected prospectively. The study was approved by the ethics committee of the Medical Faculty (anonymized) (approval number D 484/17). Written informed consent was obtained from the attendees for participation in the study and further processing of the data for publication.

**Figure 1:**
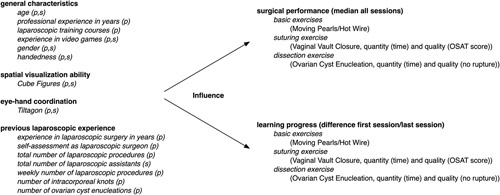
Schematic illustration of the study concept; s=students, p=physicians.

The impact of general characteristics, previous laparoscopic experience, spatial visualization ability, and eye–hand coordination on the surgical performance and learning progress of the participants was analyzed. We present the concept of the course, the methods of recording personal characteristics, and finally the statistical analysis. Figure 1 shows the study design.

**Figure 2:**
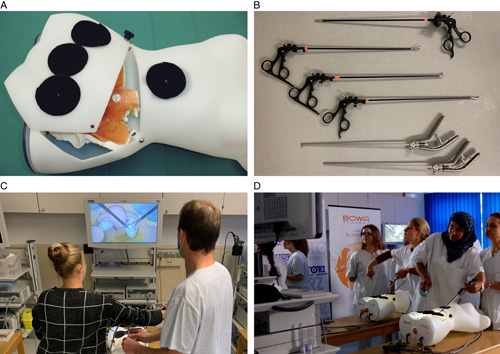
Training setup. (A) The pelvitrainer Realsimulator 2.0. (B) Laparoscopic instruments. (C) Laparoscopic setup and imaging system. (D) Attendees during a training course. From Ackermann *et al*.^15^. Reproduced with kind permission by *International Journal of Surgery*.

**Figure 3:**
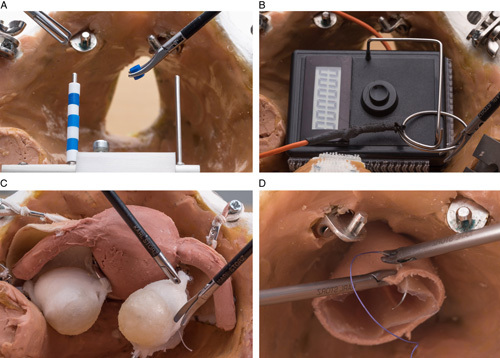
Laparoscopic exercises. (A) Moving Pearls. (B) Hot Wire. (C) Vaginal Vault Closure. (D) Ovarian Cyst Enucleation. From Ackermann *et al*.^15^. Reproduced with kind permission by *International Journal of Surgery*.

The exercises on the pelvitrainer were performed on the Realsimulator 2.0 (Endodevelop, Saarbrücken Pelvic School, Saarbrücken, Germany). Figure 2 shows the pelvitrainer and the training setup. It consisted of a multilevel training concept with two basic exercises (*Moving Pearls* and *Hot Wire*) and two advanced exercises (*Vaginal Vault Closure* and *Ovarian Cyst Enucleation*). The training concept and setting have been described in detail in a previous work^15^. Briefly, the *Moving Pearls* exercise required that the participant move eight pearls from one metal stick to another and back. Success was measured in the time taken per pearl. In the *Hot Wire* exercise, a metal ring had to be passed over a metal wire without touching it. Success was measured in time per contact. In the *Vaginal Vault Closure* exercise, a suture of a vaginal closure after hysterectomy had to be performed by two intracorporeal single knots. Success was determined first by recording time (quantity), and second by the Objective Structured Assessment of Technical Skill (OSAT) score (quality). The last exercise called for *Ovarian Cyst Enucleation* without cyst rupture in an ovarian model. Success was measured by the time taken for enucleation without cyst rupture. The four exercises are shown in Figure 3. The basic exercises were performed three times, and the advanced exercises were performed twice. Surgical performance was determined by the median of all sessions. Learning progress was defined as the difference between the first and the last session.

The attendees were asked to fill a questionnaire addressing general features (age, gender, handedness, professional experience in years, experience in video games) and *previous laparoscopic experience* (experience in laparoscopic surgery in years, self-assessment as a laparoscopic surgeon, total number of laparoscopic procedures, weekly number of laparoscopic procedures, number of intracorporeal knots, number of ovarian cyst enucleations). An analog nominal scale (ANS) was used to query self-assessment in regard of experience in video games and self-assessment as a laparoscopic surgeon. The questionnaire for the students was adapted to the corresponding preconditions. Data such as age, gender, handedness, and the number of laparoscopic surgeries as an assistant were inquired. Students were also asked to provide a self-assessment of their video game skills.

To assess spatial visualization, all participants completed the *Tube Figures Test* from the German Test for Medical Studies (TMS)^16^. The TMS and each exercise included in the test have been scientifically studied and confirmed over several years in a large number of participants with regard to prediction of the respective investigated trait. The tube figures test is particularly well suited to determine spatial visualization ability^16^. Here, two two-dimensional images of a transparent cube with differently arranged tubes are shown. The participant must indicate, on the second picture, the aspect or side (right, left, below, above, back) from which the cube is shown in the second illustration. Twenty-four figures of tubes had to be evaluated in 15 min. The exercise was explained by an example before the attendees took the test.

To test their eye–hand coordination skills, all participants had to play the tablet game Tiltagon^17^. A ball had to be guided to the target on a changing surface by tilting the tablet without letting the ball fall to the ground. The distance successfully covered was given in percent (0–100%). The participants completed a total of 15 runs of the game. The test was selected (J.B. and J.P.) after extensive web-based research (J.A., I.A., L.M., N.M., and G.P.). Similar to laparoscopy, a three-dimensional image is shown on a two-dimensional screen and the player also works with both hands. The simple score allows further statistical use of the training results. A scientific validation of the test did not exist at that time. At the time of study initiation, no similarly functioning validated test for eye–hand coordination could be found despite extensive web-based searching.

The IBM SPSS Statistics 23 program was used for statistical analysis. Variables were checked for normal distribution with the Kolmogorov–Smirnov test. Wilcoxon’s test for paired differences was used for non-normally distributed data. The Mann–Whitney *U* test was used for pairwise comparisons without normal distribution. Spearman’s rho test was used for correlation analysis when significant deviations from normal distribution were found. Tests were performed bilaterally and a significance level of 5% was used (*P*<0.05). For better visualization of the correlation coefficient *r*, the results were presented visually in the form of correlation plots.

Regarding the evaluation of correlation coefficients, it should be noted that positive or negative correlations make different statements depending on the exercise. For *Moving Pearls*, *Hot Wire*, *Vaginal Vault (quantity)* and *Ovarian Cyst*, a negative correlation with the attendee’s general characteristics, better eye–hand coordination, better spatial visualization ability, or increasing *previous laparoscopic experience* expresses a correlation with better surgical performance. For *Vaginal Vault (quality)*, a positive correlation expresses correlation with better surgical performance. The opposite is true of learning progress. Therefore, the correlation coefficients are given here and in the following without a +/− sign. In the figures, however, positive correlations are marked in blue and negative correlations in red.

Our study design is based on an explorative approach in order to find out the best possible factors influencing surgical performance and learning progress. Therefore, initially, only single values of the correlations were considered. To provide a possible outlook for further research, a multiple factor analysis was connected. For this purpose, a multiple linear regression analysis was performed if more than one significant influencing factor (*P*<0.05, *r*>0.2) was present in the training result or the learning success of the respective exercises.

## Results

A total of 206 physicians from 21 nations participated in the courses. Of these, 180 (success rate/response rate 87.4%) successfully completed the multilevel training concept. The majority of the participants were gynecologists (91.7%), followed by general surgeons (6.1%) and urologists (2.2%). Specialists or those with higher expertise accounted for 53.9%, and residents, 44.4%. As a comparative group, 261 students in their fifth year of medical school completed the same course as part of their clinical internship in gynecology (success rate/response rate: 100%). Sociodemographic data and differences between the two groups are summarized in Table 1.

**Table 1:** Description of the attendees.

	Physicians	Medical students	
Number of filled questionnaires (attendees, response rate)	180 (206, 87.4%)	261 (261, 100%)	*P*<0.001
Age (median)	36 years (range 26–78 years)	25 years (range 21.46 years)	*P*<0.001
Gender			n.s.
Female	126 (70.0%)	168 (64.4%)	
Male	54 (30.0%)	93 (35.6%)	
Handedness			*P*<0.05
Right	178 (98.9%)	245 (92.8%)	
Left	2 (1.1%)	16 (6.1%)	
Both	0	3 (1.1%)	
Number of endoscopy training courses attended in the past (median)	1 (range 0–50)	n/a	n/a
Specialization			
Gynecology	165 (91.7%)	n/a	n/a
Urology	4 (2.2%)	n/a	n/a
General surgery	11 (6.1%)	n/a	n/a
Professional experience (median)	6.75 years (range 0–34 years)	n/a	n/a
Professional status			
Resident	80 (44.4%)	n/a	n/a
Specialist	72 (40.0%)	n/a	n/a
Consultant	25 (13.9%)	n/a	n/a
Not specified	3 (1.7%)	n/a	n/a
Number of laparoscopic surgeries as a surgeon (median)	25 (range 0–5000)	n/a	n/a
Number of laparoscopic surgeries as an assistant (median)	60 (range 0–3000)	0 (range 0–30)	*P*<0.001

**Figure 4:**
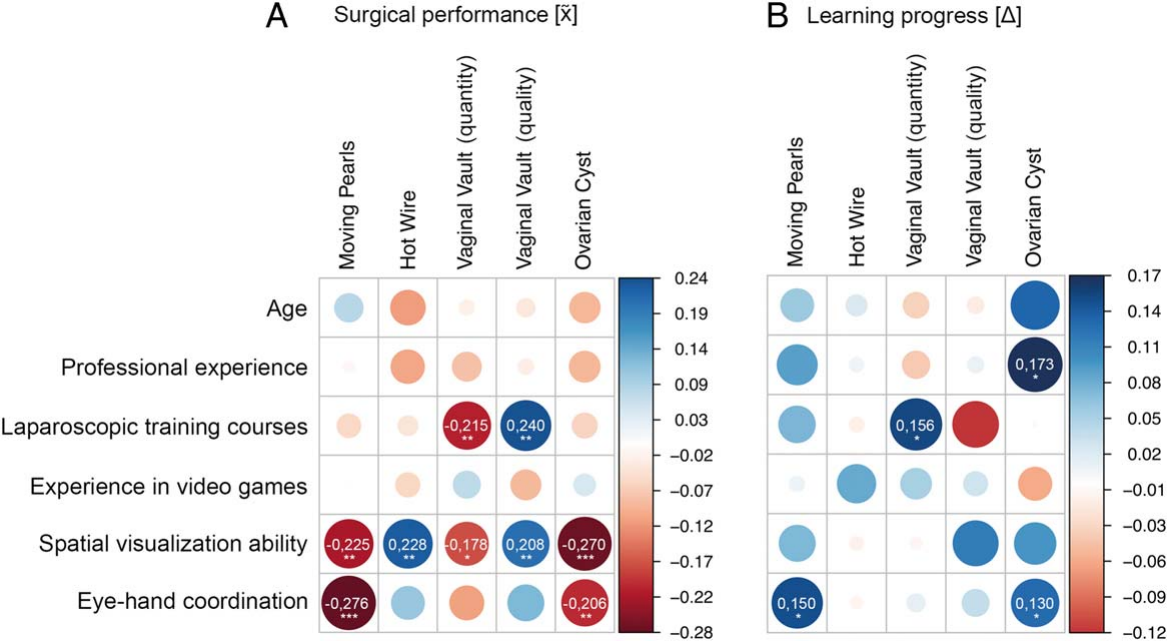
Correlations in *r* of general characteristics, spatial visualization ability, and eye–hand coordination of the physician participants on (A) surgical performance and (B) learning progress. The thicker the dot, the stronger is the correlation. Positive correlations are shown in blue and negative correlations in red. In case of significant correlation, the correlation coefficient *r* is indicated. Significant correlations are coded as follows: *P*<0.05 (*), *P*<0.01 (**), *P*<0.001 (***). However, it should be noted that a negative and a positive correlation express different conditions, depending on the exercise and the participant characteristics. For *Moving Pearls*, *Hot Wire*, *Vaginal Vault (quantity)*, and *Ovarian Cyst*, a negative correlation with characteristics (*age, years of professional experience, number of laparoscopic training courses, experience in video games, spatial visualization ability, and eye*–*hand coordination*) is correlated with better surgical performance. For *Vaginal Vault (quality)*, a positive correlation with characteristics expresses a correlation with better surgical performance (A). For learning progress, the opposite is true (B).

To examine the impact of general characteristics, spatial visualization ability, and eye–hand coordination on the surgical performance of the physicians, we performed correlation analyses between these factors and determined the respective median training score for the individual exercises (see Fig. 1). The strongest correlations with good surgical performance were noted for a high score in the *Tube Figures Test* (spatial visualization ability, *r*=0.18–0.27, *P*<0.01, significant in all exercises), followed by a high score in the tablet game *Tiltagon* (eye–hand coordination, *r*=−0.10/0.28, significant correlations only for Moving Pearls and Ovarian Cyst, *P*<0.01). The number of previously attended laparoscopic training courses were significantly correlated only with the suturing exercise *Vaginal Vault Closure* (*r*=0.22/0.24, *P*<0.01). Age, professional experience in years, and experience in video games had no significant impact on surgical performance. Figure 4A shows the correlation coefficient *r* for each exercise in the form of a correlation plot visually. No statement could be made about the impact of handedness on the training result because a mere two attendees (1.1%) were left-handed. The impact of gender was also not investigated because genders were unequally distributed in the group (large number of inexperienced and small number of experienced female participants).

To examine the impact of general characteristics, spatial visualization ability, and eye–hand coordination on learning progress, we performed correlation analyses between these factors and the respective difference between the first and last training session (see Fig. 1). Overall, the impact on learning progress was less strong and more diversely distributed than the impact on surgical performance. The strongest correlations were noted for years of experience and *Ovarian Cyst Enucleation* (*r*=0.17, *P*<0.05), followed by the number of training courses in laparoscopic surgery and *Vaginal Vault Closure* (*r*=0.16/0.12, *P*<0.05). Also, there were significant correlations between the ability of eye–hand coordination (*Tiltagon*) as well as *Moving Pearls* and *Ovarian Cyst Enucleation* (*r*=0.15/0.13, *P*<0.05). Figure 4B shows the correlation coefficient *r* for each exercise in the form of a correlation plot.

**Figure 5:**
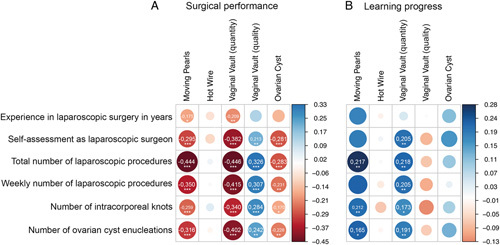
Correlations in *r* of prior experience of the physician participants on (A) surgical performance and (B) learning progress. The thicker the dot, the stronger is the correlation. Positive correlations are shown in blue and negative correlations in red. In case of significant correlation, the correlation coefficient *r* is indicated. Significant correlations are coded as follows: *P*<0.05 (*), *P*<0.01 (**), *P*<0.001 (***). However, it should be noted that a negative and a positive correlation express different conditions, depending on the exercise and participant characteristics. For *Moving Pearls*, *Hot Wire*, *Vaginal Vault (quantity)*, and *Ovarian Cyst*, a negative correlation with increasing *participants experience* expresses a correlation with better surgical performance. For *Vaginal Vault (quality)*, a positive correlation with increasing *participants experience* expresses a correlation with better surgical performance (A). For learning progress, the opposite is true (B).

To investigate the impact of specific previous laparoscopic experience on the surgical outcome, we performed correlation analyses between prior experience and the respective median training score of each exercise (see Fig. 1). The strongest correlation with good surgical performance was noted for a large absolute number of laparoscopic procedures (*r*=0.28–0.45, *P*<0.001, no correlation for *Hot Wire*). The weakest correlation with good training results was noted for experience in laparoscopic surgery in years. No correlation at all was seen between previous laparoscopic experience and performance in the *Hot Wire* exercise. In Figure 5A, the correlation coefficient *r* for the respective exercises is shown visually in the form of a correlation plot.

Previous laparoscopic experience was more strongly correlated with learning progress than with general characteristics, spatial visualization ability, and eye–hand coordination. Overall, weaker correlations were noted for learning progress than for surgical performance.

With regard to learning progress, the strongest correlations were noted with previous laparoscopic experience. However, this correlation was not as strong as that between surgical performance and previous laparoscopic experience. Analogous to surgical performance, the strongest correlation was noted for learning progress and the absolute number of previous surgeries (r=0.1-0.28, *P*<0.05, no correlation for *Hot Wire*). No correlation at all was observed between previous laparoscopic experience and learning progress in the Hot Wire exercise (Fig. 5B).

**Figure 6:**
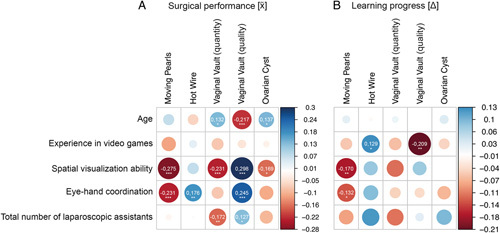
Correlations in *r* of personal factors of the student participants on (A) surgical outcome and (B) learning success. The thicker the dot, the stronger is the correlation. In case of significant correlation, the correlation coefficient *r* is indicated. Significant correlations are coded as follows: *P*<0.05 (*), *P*<0.01 (**), *P*<0.001 (***). However, it should be noted that a negative and a positive correlation express different conditions, depending on the exercise and participant characteristics. For *Moving Pearls*, *Hot Wire*, *Vaginal Vault (quantity)*, and *Ovarian Cyst*, a negative correlation with participant characteristics (*age, experience in video games, spatial visualization ability, eye*–*hand coordination*, and *total number of laparoscopic assistants*) expresses a correlation with better surgical performance. For *Vaginal Vault (quality)*, a positive correlation with participant characteristics expresses a correlation with better surgical performance (A). For learning progress, the opposite is true (B).

Among students, the strongest correlations with good surgical performance were noted for a high spatial visualization ability (*Tube Figures Test*) in all of the exercises (*r*=0.17–0.30, *P*<0.001) except Hot Wire (*r*=0.07, *P*=0.24). The second-strongest correlations were observed for good hand–eye coordination [*Tiltagon*, *Moving Pearls* (0.23, *P*<0.001), *Hot Wire* (0.18, *P*<0.01), and *Vaginal Vault Closure* (*r*=0.25, *P*<0.001)]. Video game experience revealed no significant correlation with surgical performance. In contrast to physicians, age also had an effect on surgical performance. Increasing age was associated with longer operating times (*r*=0.13, *P*<0.05) and a lower OSAT score (*r*=0.22, *P*<0.001) in the laparoscopic suturing exercise (*Vaginal Vault Closure*). In the more complex exercise of *Ovarian Cyst Enucleation* as well, increasing age was associated with significantly longer operating times (*r*=0.14, *P*<0.05). Likewise, students with previous experience in laparoscopic assistance performed better in the more complex exercise of *Vaginal Vault Closure* (quality/quantity, *r*=0.13/0.17, *P*<0.05/0.01). Handedness and gender had no impact on the training result. In Figure 6A, the correlation coefficient *r* for the respective exercises is shown in the form of a correlation plot.

Students also showed lower correlations between the studied parameters with regard to learning progress. Interestingly, stronger correlations were seen here for prior experience in video games (hot wire: *r*=0.13, *P*<0.05; vaginal vault OAST: *r*=0.21; *P*<0.001). Good spatial visualization abilities and eye–hand coordination were significantly correlated with learning progress in the basic exercise of *Moving Pearls* (*r*=0.17/0.13, *P*<0.01/0.05). Age, handedness, and gender had no impact on learning progress (see Fig. 6B). All correlation analyses including correlation coefficient *r* and significance level are presented in Table 2.

**Table 2:** Correlations in *r* of general characteristics, spatial visualization ability, and eye–hand coordination.

	Moving Pearls		Hot Wire		Vaginal Vault Closure, quantity (time)		Vaginal Vault Closure, quality (OSAT score)		Ovarian Cyst Enucleation	
	*Performance*	*Learning progress*	*Performance*	*Learning progress*	*Performance*	*Learning progress*	*Performance*	*Learning progress*	*Performance*	*Learning progress*
*Physicians*										
** **Age										
** ** *r*	0.077	0.044	−0.115	−0.059	0.022	−0.039	−0.032	−0.016	−0.089	0.138
** ** *P*	0.303	0.560	0.141	0.453	0.775	0.608	0.668	0.839	0.260	0.068
** ** *n*	179	174	166	165	179	179	179	179	163	163
** **Professional experience in years										
** ** *r*	−0.011	0.040	−0.107	−0.012	−0.080	−0.044	−0.023	0.015	−0.091	**0.173**
** ** *P*	0.884	0.600	0.167	0.883	0.288	0.559	0.763	0.839	0.247	**0.021**
** ** *n*	180	175	166	166	180	180	180	180	164	**164**
** **Laparoscopic training courses										
** ** *r*	−0.053	0.057	−0.037	−0.013	**−0.215**	0.156	**0.240**	−0.124	−0.060	0.002
** ** *P*	0.484	0.456	0.643	0.867	**0.004**	0.037	**0.001**	0.099	0.446	0.984
** ** *n*	179	179	167	166	**179**	179	**179**	179	164	164
** **Experience in video games										
** ** *r*	−0.002	0.011	−0.056	0.087	0.070	0.056	−0.086	0.034	−0.045	−0.065
** ** *P*	0.974	0.888	0.475	0.264	0.354	0.453	0.252	0.650	0.565	0.390
** ** *n*	179	179	167	166	179	179	179	179	164	164
Tube figures (spatial visualization ability)										
** ** *r*	**−0.225**	0.020	**0.228**	0.022	**−0.178**	−0.008	**0.208**	**0.120**	**−0.270**	−0.076
** ** *P*	**0.002**	0.715	**0.003**	0.694	**0.017**	0.885	**0.005**	**0.031**	**0.000**	0.180
** ** *n*	**180**	175	**167**	166	**180**	180	**180**	**180**	**164**	178
Tiltagon (eye–hand coordination)										
** ** *r*	**−0.276**	**0.150**	0.104	−0.017	−0.111	0.019	0.124	0.043	**−0.206**	0.134
** ** *P*	**0.000**	**0.012**	0.182	0.760	0.139	0.712	0.098	0.412	**0.008**	0.032
** ** *n*	**180**	**175**	167	166	180	180	180	180	**164**	164
Experience in laparoscopic surgery in years										
** ** *r*	**−0.175**	0.096	−0.068	−0.018	**−0.209**	0.045	0.137	0.005	−0.148	0.118
** ** *P*	**0.019**	0.207	0.387	0.832	**0.005**	0.551	0.068	0.943	0.059	0.116
** ** *n*	**179**	179	166	165	**179**	179	179	179	163	163
Self-assessment as laparoscopic surgeon										
** ** *r*	**−0.295**	0.145	−0.089	−0.016	**−0.383**	0.205	**0.213**	−0.092	**−0.281**	0.162
** ** *P*	**0.000**	0.056	0.262	0.836	**0.000**	0.007	**0.005**	0.228	**0.000**	0.075
** ** *n*	**174**	174	162	162	**174**	174	**174**	174	**160**	160
Total number of laparoscopic procedures										
** ** *r*	**−0.444**	0.217	0.030	0.008	**−0.446**	0.218	**0.326**	−0.077	**−0.283**	0.099
** ** *P*	**0.000**	0.004	0.701	0.921	**0.000**	0.003	**0.000**	0.307	**0.000**	0.190
** ** *n*	**179**	175	167	166	**179**	179	**179**	179	**164**	164
Weekly number of laparoscopic procedures										
** ** *r*	**−0.350**	0.147	0.012	−0.030	**−0.415**	0.205	**0.307**	−0.101	**−0.243**	0.025
** ** *P*	**0.000**	0.053	0.878	0.701	**0.000**	0.006	**0.000**	0.179	**0.002**	0.744
** ** *n*	**178**	177	167	166	**178**	178	**178**	178	**163**	177
Number of intracorporal knots										
** ** *r*	**−0.259**	0.212	0.060	−0.074	**−0.340**	**0.173**	**0.284**	−0.132	**−0.170**	0.091
** ** *P*	**0.000**	0.005	0.440	0.342	**0.000**	**0.021**	**0.000**	0.077	**0.030**	0.228
** ** *n*	**179**	175	167	167	**179**	**179**	**179**	179	**164**	164
Number of ovarian cyst enucleations										
** ** *r*	**−0.316**	**0.165**	−0.025	−0.031	**−0.402**	**0.191**	**0.242**	−0.064	**−0.228**	0.131
** ** *P*	**0.000**	**0.029**	0.748	0.691	**0.000**	**0.010**	**0.001**	0.393	**0.003**	0.082
** ** *n*	**179**	**179**	167	166	**179**	**179**	**179**	179	**164**	163
*Students*										
** **Age										
** ** *r*	0.069	0.023	−0.064	−0.005	**0.132**	0.016	**−0.217**	−0.001	**0.137**	0.030
** ** *P*	0.264	0.708	0.306	0.942	**0.034**	0.802	**0.000**	0.993	**0.039**	0.649
** ** *n*	261	261	261	261	**260**	258	**260**	250	**228**	228
** **Experience in video games										
** ** *r*	−0.117	−0.055	0.035	**0.129**	−0.080	−0.063	−0.039	**−0.209**	−0.022	−0.045
** ** *P*	0.059	0.375	0.572	**0.038**	0.194	0.322	0.534	**0.001**	0.738	0.496
** ** *n*	261	261	261	**261**	260	258	260	**250**	228	228
** **Tube figures (spatial visualization ability)										
** ** *r*	**−0.275**	**−0.170**	−0.073	−0.078	**−0.231**	0.115	**0.298**	0.076	**−0.169**	−0.001
** ** *P*	**0.000**	**0.000**	0.241	0.207	**0.000**	0.066	**0.000**	0.233	**0.010**	0.987
** ** *n*	**261**	**261**	261	261	**260**	258	**260**	250	**228**	228
** **Tiltagon (eye–hand coordination)										
** ** *r*	**−0.231**	**−0.132**	**0.176**	0.089	−0.080	−0.047	**0.245**	−0.039	−0.111	−0.071
** ** *P*	**0.000**	**0.033**	**0.004**	0.152	0.200	0.448	**0.000**	0.537	0.096	0.285
** ** *n*	**261**	**261**	**261**	261	260	258	**260**	250	228	228
** **Number of laparoscopic surgeries as assistant										
** ** *r*	−0.017	−0.085	0.005	0.121	**−0.172**	−0.112	0.127	**0.027**	−0.097	0.093
** ** *P*	0.784	0.169	0.938	0.050	**0.005**	0.072	0.040	**0.669**	0.146	0.160
** ** *n*	261	261	261	261	**260**	250	260	**250**	228	228

significant correlations are marked bold.

OSAT, Objective Structured Assessment of Technical Skill.

The multiple factor analysis showed a partially different weighting of the influence of the individual factors than in the single factor analysis. In the group of physicians, the (1) hand–eye coordination (*P*=0.003) and (2) spatial visualization ability (*P*=0.009) remained as independent influencing factors for the surgical performance in the *Moving Pearls* exercise in the linear regression analysis. For the *Vaginal Vault Closure (quantity)* exercise, (1) self-assessment as laparoscopic surgeon (*P*=0.007) and (2) weekly number of laparoscopic procedures (*P*=0.02) emerged as the strongest independent influencing factors. For *Vaginal Vault Closure (quality)*, these were (1) weekly number of laparoscopic procedures (*P*<0.001) and (2) spatial visualization ability (*P*=0.001). For the *Ovarian Cyst Enucleation* exercise, (1) self-assessment as laparoscopic surgeon (*P*=0.001) and (2) spatial visualization ability (*P*=0.006) remained as independent influencing factors. The results including the excluded influencing factors are shown in Table 3.

**Table 3:** Multiple factor analysis of participants characteristics (linear regression).

Dependent variable	Predictor variables	Excluded variables
*Physicians*		
Moving Pearls	Eye–hand coordination (*P*=0.003) Spatial visualization ability (*P*=0.009)	Self-assessment as laparoscopic surgeon Total number of laparoscopic procedures Weekly number of laparoscopic procedures Number of intracorporal knots Number of ovarian cyst enucleations
Vaginal Vault Closure (quantity)	Self-assessment as laparoscopic surgeon (*P*=0.007) Weekly number of laparoscopic procedures (*P*=0.02)	Laparoscopic training courses Experience in laparoscopic surgery in years Total number of laparoscopic procedures Number of intracorporal knots Number of ovarian cyst enucleations
Vaginal Vault Closure (quality)	Weekly number of laparoscopic procedures (*P*<0.001) Spatial visualization ability (*P*=0.001)	Laparoscopic training courses Self-assessment as laparoscopic surgeon Total number of laparoscopic procedures Number of intracorporal knots Number of ovarian cyst enucleations
Ovarian Cyst Enucleation	Self-assessment as laparoscopic surgeon (*P*=0.001) Spatial visualization ability (*P*=0.006)	Eye–hand coordination Total number of laparoscopic procedures Weekly number of laparoscopic procedures Number of ovarian cyst enucleations
*Students*		
Moving Pearls	Eye–hand coordination (*P*=0.005) Spatial visualization ability (*P*=0.005)	–
Vaginal Vault Closure (quality)	Eye–hand coordination (*P*<0.001) (3/6) Age (*P*=0.001) Spatial visualization ability (*P*=0.002) (4/6)	–

In the student group, only the *Moving Pearls* and *Vaginal Vault Closure (quality)* exercises met the criteria for multiple factor analysis. For the *Moving Pearls* exercise, the results for (1) hand–eye coordination (*P*=0.005) and (2) spatial visualization ability (*P*=0.005) remained as independent influencing factors, as was already the case for the physicians. For the *Vaginal Vault Closure (quality)* exercise, also as for the physicians, the result for (1) hand–eye coordination (*P*<0.001) and (3) spatial visualization ability (*P*=0.002) and additional (2) age (*P*=0.001) remained. Interestingly, no influencing factor was excluded in the undergraduate group. The results are presented in Table 3.

## Discussion

In view of the ongoing development of surgical methods, the persistent scarcity of resources in the health-care sector, and consistent efforts to improve patient safety, it would be desirable to have tools that would permit early and accurate selection of medical students and young physicians as prospective surgeons^18^. However, our knowledge as to what makes a good surgeon or even a talented surgeon is limited^19,20^. Any development of decision aids or selection procedures should be preceded by a clear concept as to whether selection is possible at all and what criteria one may use to predict a competent surgeon. The aim of this work was to investigate those factors that influence surgical performance as well as the learning ability of surgeons and medical students.

The strongest correlation, both for good surgical performance and good learning progress, was noted for the total number of laparoscopic surgeries performed as a surgeon. The impact of this factor was greater than that of the weekly number of operations as a surgeon or the years of experience in minimally invasive surgery. This observation confirms a previous statement to the effect that the absolute number of operations, that is the quantitative experience of a surgeon, is a good predictor of surgical success^7,8,21,22^. This is also reflected in many certification programs, which prescribe the absolute number of specific surgeries as a defining requirement^23^. Interestingly, in the multiple factor analysis, the weekly number of surgeries proved to be a stronger independent parameter than the total number. One explanation for this could be the two different mathematical calculation models. In the multiple factor analysis, a linear regression is performed; however, a correlation does not necessarily have to be linear (as is also the case for the total number of laparoscopic surgeries). It is likely that both factors describe different aspects of surgical experience, with the total number describing more experience and the weekly number describing routine. Perhaps both would need to be used to best predict surgical success. To obtain further clarification of this issue, further studies with an appropriate design should be chosen. With our present study, this is not conclusively possible.

The fact that experience is one of the most important predictors of surgical success could be used as a prime argument against the selection of surgeons based on specific characteristics and talents. Any surgeon will be able to enhance the quality of his/her work by performing more numerous operations. However, based on traditional training concepts, the quality of performance is also determined by a substantial number of operations on living patients. This approach is contradicted by concerns about patient safety and limited resources in the health-care system. This is especially true in view of the availability of alternative models. Two different approaches could be used to resolve the problem: (a) Selection criteria for particularly suitable or talented physicians and only their admission to further training as surgeons. (b) Allowing no aspirant to become a surgeon until he/she has achieved a certain quality threshold, for example, on a surgical simulator (equivalent to pilot training^24,25^), no matter how many passes the aspirant needs to achieve this level of quality. However, which of the two options, or even a combination of the two, would be the most suitable approach, cannot be stated at the present time. On the one hand, we are faced with a paucity of valid selection criteria. On the other hand, we do not know which training methods would be suitable for achieving the appropriate level of surgical skill.

Notably, the subject has been given scarce attention in current health policy discussions. An actual advancement in the field would require more strict statutory guidelines and the development of adequate training devices and nation-wide training programs. However, our research disclosed new options to overcome the individual learning curve through appropriate training with devices in the field of minimally invasive surgery (pelvic trainers, virtual trainers, training on animals, and training on cadavers)^26–29^.

The results of the exercises for testing spatial visualization ability and eye–hand coordination had a relatively strong impact on surgical performance. In particular, the *Tube Figures Test*, which is also used in the German medical student selection test (TMS), showed a strong correlation with surgical performance. Also in the multiple factor analysis, the results in the two tests remained as constant predictors (tube figures test in 4 of 6 and eye–hand coordination in 3 of 6 analyses) on surgical performance. This is particularly significant because the eye–hand coordination tiltagon test had not previously received validation as a test for a related question. Furthermore, it confirms the predictive power of such tests regarding surgical performance. This shows that: (a) Endoscopic training in surgery must follow a gradient scale of complexity (from easy to difficult) and simultaneously train individual skills (spatial visualization, hand–eye coordination, etc.) selectively. (b) It appears possible to select talented surgeons by special selection procedures. (c) Making handicaps individually visible, a widely used approach in professional sports, might enable us to provide individual training^30,31^.

Age had no significant impact on surgical performance or learning progress among physicians. Interestingly, among students, we noted a weak impact of age on surgical performance. Predictably, the median age of students was significantly lower. This might possibly confirm the hypothesis that younger age may generally be expected to result in better learning ability and adaptability^32^. It is also an argument in favor of imparting surgical skills early to medical students as well as surgeons. It should be noted that our study design did not have a specific focus on age as a predictor of surgical success or learning progress. To make a true and more accurate statement would require appropriate randomization (constant age distribution with similar characteristics and test scores of participants in each case), which was not performed here.

In our study, the number of laparoscopic training courses attended previously by the participants had a positive impact on the intracorporeal suture exercise (*Vaginal Vault Closure*). This observation confirms the thesis that complex surgical techniques can be practiced very well on the pelvitrainer or other virtual trainers^33^. It also confirms our notion that laparoscopic training courses focus excessively on suturing exercises. In the future, it would be advisable to give greater attention to the use of innovative laparoscopic trainers and training concepts incorporating technical developments in terms of training tools^15,34,35^. Surgical training courses are not a mandatory part of nation-wide resident programs in Germany. Such training courses do exist as a standardized concept in the German Endoscopic Gynecological Society^23^. In general surgery, however, a surgeon may become an expert and a specialist in endoscopic surgery without having attended a specific training course in endoscopy or followed an official training concept in the sub-specialty. The German Surgical or Urological Society provides no standardized program of this nature.

Our study has some limitations that need to be addressed in further studies. For example, there was an uneven distribution of the different disciplines (the majority of the participating physicians were gynecologists). An even randomization would have been desirable. In principle, however, the specialist discipline represents only a very rough criterion of a physician practicing surgery, since there are large overlaps for all three specialties. Thus, laparoscopies are also performed in the gynecologic and urologic fields by general surgeons in many countries. And gynecologists and urologists sometimes perform the same operations (e.g. pelvic floor and incontinence surgery).

In the present study, the impact of the individual factors on overall learning progress was less pronounced than it was on surgical performance. The main reason may have been the small number of repetitions (two or three runs) in our training concept. Although a significant improvement in training scores was observed for all of the exercises performed by the attendees, no learning curve can be replicated with a mere three runs^15,36^. On the other hand, factors other than those investigated here could have a stronger impact on learning abilities. We need further investigations with significantly more repetitions, additional and diverse pretests, in order to make definitive statements. For example, the influence of individual characteristics of visual and auditory perception, long-term and short-term memory, previous knowledge of anatomical conditions and surgical methods, experience in the field of open surgery, and certainly the motivation of the individual participants should be investigated.

Notably, the absolute correlation coefficients shown are relatively low. Further tests will be needed to make a robust statement. We know from scientific research in psychology that correlations between test results and personal characteristics are usually low in a specific population and quite often significantly lower than the correlation coefficients reported in the present investigation^37–39^.

The approach of our study was exploratory. In the form of a screening, we aimed to uncover possible factors influencing surgical performance and learning progress of operating physicians. In addition, a multiple factor analysis was performed. This was used to reselect the personality traits of the participants that appeared to have an influence. However, this procedure should not be understood as an actual exclusion of a large number of potential influencing factors, but rather as an outlook. The study design was not designed for final selection of the factors studied. This will have to be done in future work based on our data, which will focus on the characteristics highlighted here.

In summary, the present work investigates factors influencing surgical performance and learning progress in a prospective, multicenter, multinational, and interdisciplinary setting. We conclude that individual factors do have an impact on surgical performance and learning ability. Further research will be needed to determine whether specific tests and selection procedures can be used to select or guide prospective surgeons. In addition, it will be necessary to determine those training measures that would help to overcome the individual learning curve and permit the best possible patient care. Health policy structures would then have to be created in order to implement such training programs in medical schools and residency training. A part of the surgical training could be held at special training centers on simulators, away from the actual patient, and thus reduce the need for training on live patients. The present report is intended to improve surgical education and optimize the level of hands-on clinical patient care in the interest of all involved parties.

## Ethical approval

The study was approved by the ethics committee of the Medical Faculty of Kiel University (approval number D 484/17).

## Consent

Written informed consent was obtained from the attendees for participation in the study and further processing of the data for publication.

## Sources of funding

The study had no main sponsor. Provision of technical equipment: Karl Storz, Olympus Medical Systems, Richard Wolf GmbH, BOWA-electronic, and Johnson & Johnson Medical. There was no external involvement in the collection, analysis, and interpretation of data.

## Author contribution

J.A.: conceptualization, data curation, formal analysis, investigation, methodology, project administration, supervision, validation, visualization, and roles/writing – original draft; J.B., J.Pahls, B.H., G.N., and L.M.: conceptualization, investigation, and writing – review and editing; J.Pape: data curation, formal analysis, resources, and software; Z.R.: writing – review and editing; C.S.; M.A., I.M.-H., A.W., and V.G.: investigation and writing – review and editing; G.P. and D.W.: investigation; S.B.: data curation and writing – review and editing; N.M.: resources, supervision, and writing – review and editing; I.A.: conceptualization, funding acquisition, investigation, methodology, project administration, resources, supervision, validation, visualization, and roles/writing – original draft.

## Conflicts of interest disclosure

Carolin Spüntrup is the owner of Endodevelop. She is responsible for the development and distribution of the pelvitrainer Realsimulator 2.0 used in this work. The associated devices and consumables were purchased at regular prices from the training centers. None of the authors received any financial benefit. All other authors declare no conflicts of interest.

## Research registration unique identifying number (UIN)

Not applicable due to observational study with physicians/trainees.

## Guarantor

Johannes Ackermann and Ibrahim Alkatout accept full responsibility for the work and the conduct of the study.

## Data availability statement

In order to minimize the possibility of unintentionally sharing information that could be used to re-identify private information, a subset of the data generated for the study can be provided by the corresponding author after written contact via E-mail to kiel.school@uksh.de.

## Provenance and peer review

The paper was not invited.

## Acknowledgement

The authors thank Saskia Struck for administrative management, Sujata Wagner of Medical Translation for professional English editing, and the following companies for their kind provision of technical equipment: Karl Storz, Olympus Medical Systems, Richard Wolf GmbH, BOWA-electronic, and Johnson & Johnson Medical.

Maximum effort has been made to avoid unintentional disclosure of data that could be used to identify individual persons; a subset of the data generated for the study can be provided by the corresponding author in writing from kiel.school@uksh.de.

The data have not been stored in a public repository.

No preregistration exists for the studies reported in this article.

We acknowledge financial support by DFG within the funding program Open Access publication costs.
